# Commentary on neoadjuvant therapy followed by local excision and two-stage total mesorectal excision: a new strategy for sphincter preservation in locally advanced ultra-low rectal cancer

**DOI:** 10.1093/gastro/gou024

**Published:** 2014-05-07

**Authors:** Andrew P. Zbar

**Affiliations:** Department of Surgery and Transplantation, Chaim Sheba Medical Center, Sackler Medical School, Tel Aviv University, Ramat Gan 52621 and Assia Medical Group, Barzel Street 10, Ramat Gan 52621, Israel. Tel: +972-54-980-5414; Email: apzbar1355@yahoo.com

The authors have provided a novel hybrid approach in a selected group of locally advanced ultra-low rectal cancers, using neoadjuvant chemoradiation followed by local excision in responders and a delayed Total Mesorectal Excision (TME) and restorative proctectomy, showing that medium-term survival is possible with acceptable functional outcome [1]. Although it is clear that the neoadjuvant approach has acceptable toxicity—with the majority of patients showing significant tumor downsizing and creating a better chance for sphincter preservation—the selection criteria for these patients still remain subjective. Here, the alternatives in those showing a partial clinical response (pCR) include radical resection, transanal local excision and a ‘wait and see' policy but the data are contradictory in the matter of assisting individual patient management [2]. One problem is that some data show relatively high recurrence rates in clinical complete responders when a ‘wait and see' strategy is followed, while studies are heterogeneous in their staging and inclusion criteria and there are differences in what constitutes a pCR. In this respect, there is only partial agreement between pcR and complete clinical response (cCR) cases [3]. This inconsistency of cCR diagnosis most probably also explains some reportedly high rates of local perirectal lymph node metastases in some series, which precludes either a ‘wait and see' plan of action or one combined with local excision [4].

Despite the encouraging results from Wang *et al.*, which mirror those recently reported from Beijing using a neoadjuvant approach followed by TME for distal rectal cancers, [5] the numbers are at this stage too small to result in adequate conclusions regarding this hybrid approach, where four out of nine cases still had lymph node involvement despite a partial response. Although it would appear that objective tumor shrinkage—as measured by magnetic resonance imaging (MRI) or even by barium enema—may assist in correlating with the final histological response [6], our assessment of responders who were less likely to have involved perirectal lymph nodes is still limited, where early FDG-PET responsiveness not only correlates with pathological response but also with relapse-free survival when TME is performed after neoadjuvant therapy for locally advanced cases [7]. Proof of the prognostic benefit of local excision as an interim procedure can only await the results of clinical randomized trials in which there is a standardization of cCR and pCR and its value will be affected by histological tumor type [8] with less tumor regression in mucinous variants, as well as by tumor location (anterior versus posterior tumors) [9]. The advantage of the approach by Wang *et al.* in this reported study will be that of using the local excision as a prognostic marker for response, since Borschitz *et al.* have shown a very low locoregional recurrence rate (under 2%) with a near-complete or complete pCR with local excision alone [10] and outcomes that are equivalent to those undergoing routine TME surgery [11]. Despite this approach, however, the high morbidity of a local excisional policy alone should be considered, suggesting that TEM alone remains an unacceptable policy [12].

It is at present hard to justify this ‘triple approach' by Wang and colleagues over conventional TME in distal locally advanced cases with sphincter preservation, although clearly the data are in line with the very low local recurrence rates after complete response [13]. The follow-up in these patients also needs to be comparatively long, as the median time for tumor regrowth can exceed five years [14]. The likelihood is that advances will come more from rigorous patient selection in advanced low tumors with a better definition of cCR by clinical, endoscopic and metabolic imaging, along with histological local excision, to better identify those patients most suited to a subsequent TME or to an observational policy. Further, the data supporting a ‘wait and see' policy in earlier responsive tumors, where salvage surgery may be performed for endoluminal recurrence, cannot effectively be extrapolated to those more advanced T3 or T4 tumors, in which initial nodal positivity can be high and residual nodal disease can be moderate. Locoregional recurrence in such cases will be a feature of residual local lymph node disease that would mandate a restorative TME where possible. In all of this, the best time to assess response currently remains unknown, as does the exact timing of subsequent surgery, which is being investigated by the ongoing NCT 01037049 UK trial that compares surgery at 6 and at 12 weeks after neoadjuvant therapy. In this regard, more extended periods before definitive surgery may actually permit a greater recorded pCR rate [15].

**Conflict of interest:** none declared.
